# Temperature is not a major factor in the differentiation of gonocytes into ad spermatogonia and fertility outcome in congenitally cryptorchid boys

**DOI:** 10.1186/s12610-021-00152-6

**Published:** 2022-01-10

**Authors:** Faruk Hadziselimovic

**Affiliations:** Cryptorchidism Research Institute, Children’s Day care center Liestal, 4410 Liestal, Switzerland

**Keywords:** Cryptorchidism, Gonocyte, Ad spermatogonium, Heat shock factors, Heat shock proteins, Fertility, Cryptorchidie, Gonocyte, Ad spermatogonia, Protéines de choc thermique, Fertilité

## Abstract

Spermatogenesis in mammals is a heat-sensitive developmental pathway incompatible with the typical mammalian body temperature of 37 °C. It is thought that this is the reason why the testicles of most mammalian males are outside of the body cavity, in the scrotum, where they function at approximately 33 °C. It has been suggested that the abnormally high temperature environment of cryptorchid testes may lead to impaired testicular development and adult infertility. Here, I summarize the clinical, genetic, and histological evidence that argues against temperature stress and in favor of hypogonadotropic hypogonadism as the underlying cause of adult infertility in cryptorchidism.

**Patient summary:** Infertility and an increased risk of testicular cancer in patients diagnosed with undescended testes are the consequence of a hormonal deficiency rather than temperature-induced cellular damage. Cryptorchidism therefore requires both surgical and hormonal treatment.

## Résumé

La spermatogenèse chez les mammifères adultes est. sensible à la chaleur et en conséquence incompatible avec la température corporelle typique des mammifères de 37 °C. On pense que ça soit la raison pour laquelle les testicules de la plupart des mammifères mâles se trouvent dans le scrotum à l’extérieur du corps où ils fonctionnent à environ 33 °C. Il a été suggéré que l’exposition des testicules cryptorchidés à une température anormalement élevée pourrais empêcher le développement testiculaire normal et ainsi être à l’origine de l’infertilité chez l’adulte. Je résume des données cliniques, génétiques et histologiques en faveur de l’hypogonadisme hypogonadotrope et contre le stress thermique comme cause sous-jacente de l’infertilité adulte dans la cryptorchidie.

**Résumé patient:** L’infertilité et un risque accru de développer un cancer des testicules chez les patients diagnostiqués avec un testicule non descendu, sont la conséquence d’un déficit hormonal plutôt que de dommages cellulaires induits par la température. La cryptorchidie nécessite donc un traitement à la fois chirurgical et hormonal.

## Introduction

Transient activation of the hypothalamus-pituitary-testicular axis occurs during the first months of life in a process called minipuberty, which is fundamentally important for the differentiation of gonocytes into A dark (Ad) spermatogonial stem cells and the development of the male reproductive organs. While several forms of cryptorchidism exist and have different origins, hypogonadotropic hypogonadism is the most common cause of this reproductive disorder [1]. Molecular observations support a crucial role for *PROK2* in the pathophysiology of cryptorchidism, whereby impaired *PROK2/CHD7/FGFR1/SPRY4* gene expression controlled by the regulators *NHLH2*, *EGR4*, and *PITX1* induces LH deficiency [2, 3]. Gonadotropin and testosterone deficiency or (more rarely) androgen receptor failure results in abrogated minipuberty and impaired transformation of gonocytes into Ad spermatogonia [2, 4]. The most severe cases of infertility are seen in men with a history of cryptorchidism who present with the most affected minipuberty and show the strongest impairment of gonocyte differentiation into Ad spermatogonia in both testes [5].

It is widely accepted that exposure of the adult testis to abnormally high temperatures induces germ cell loss and infertility. However, the impact of higher temperature upon the prepubertal human testis is still a matter of debate [1, 6]. Recently, it was suggested that in cryptorchid mice with genomic defects, it is the gene-dosage effect on cellular processes in the seminiferous tubules that contributes to the infertility outcome, rather than elevated temperature due to the abnormal position of the testis in the pelvis or abdomen [7]. Opposing that view, Loebenstein et al. postulated that human gonocytes normally transform into Ad spermatogonia only when the temperature in the scrotum is lower than the core body temperature [6]. The authors speculate that inadequate transformation during minipuberty, triggered by the undescended testis’ abnormally high temperature, leads to a deficient stem cell pool and infertility [6]. Furthermore, Loebenstein et al. propose that apoptosis of the remaining gonocytes is inhibited, enabling a sub-population of these cells to persist, and eventually leading to carcinoma in situ and testicular cancer during adulthood [6**].**

This article does not aim to provide a global overview of competing thoughts in the field about the molecular causes of cryptorchidism and its optimal treatment, but rather focuses on results that question the role of elevated temperature in gonocyte transformation into Ad spermatogonia during minipuberty.

## Developmental stage-specific temperature control of testis development and function

Indeed, the temperature of an undescended testis in its cryptorchid location in 13- to 180-month-old boys is significantly higher than that of the contralateral normally descended testicle (34.4 °C ± 0.9 versus 33.2 °C ± 1.2; *p* < 0.001) [8]. However, the scrotal temperature, which closely reflects the testicular temperature, is increased in boys wearing disposable plastic-lined nappies that blunt or even abolish the physiological testicular cooling mechanism without, however, affecting adult fertility [9]. Another phenomenon inconsistent with a negative effect of high temperature is associated with mutation of the *SRD5A2* gene. Affected individuals have deficient dihydrotestosterone formation, resulting in phenotypically female genitalia and bilateral cryptorchidism. In testes from boys bearing a defective allele, the numbers and differentiation of spermatogonia are normal during the entire prepubertal period, indicating that mini-puberty occurs normally. These findings are in sharp contrast to observations in boys whose testes express normal SRD5A2 enzyme levels but develop isolated bilateral cryptorchidism and have an abrogated mini-puberty, lacking Ad spermatogonia [10]. Given that the positions of the testes in both groups were identical, they must have been exposed to similar temperatures. Therefore, if temperature were the only explanation for the massive germ cell loss, the two groups should have similar germ cell populations. Since this is not the case, temperature appears to have a negligible effect on the proliferation and differentiation of Ad spermatogonia [10]. Recently, Shiraishi et al. proposed that a temperature lower than the normal mammalian body temperature is necessary for the normal development of infantile testes. The authors report that after orchidopexy testicular volume increased significantly because testes were exposed to lower temperature. However, it is unclear how important this increase of 15% is for fertility [11]. Direct effects of elevated temperature on prepubertal spermatogonia have never been proven. Importantly, there are no long-term studies on any influence of elevated temperature during prepuberty on the fertility outcome. However, in our unique long-term study (that spanned more than 20 years) we analyzed 89 cryptorchid boys between the ages of one to 16 years. (5) They were subjected to bilateral testicular biopsy at the time of orchidopexy. Seventy (78%) exhibited unilateral and 19 (22%) bilateral cryptorchidism. No hormonal treatment was performed prior to the surgery. Importantly, transformation of gonocytes / fetal and undifferentiated spermatogonia into Ad spermatogonia starts at mini-puberty and is a continuous LH-dependent process during prepubertal period. The data we obtained showed that the testes of patients in high, intermediate, and low infertility risk groups contained a lower number of Ad spermatogonia as compared to the control group. (5) In cases where Ad spermatogonia were depleted in both testes, no patient showed a normal sperm count despite successful surgery and descended gonads being exposed to lower temperature. This finding seriously questions the importance of temperature for the development of Ad spermatogonia. Furthermore, in patients with bilateral cryptorchidism having Ad spermatogonia in one or both testes, 55% developed a normal sperm count. Thus, the presence of Ad spermatogonia and not the total germ cell count is a predictor of male fertility. (5) The importance of a relative post-pubertal gonadotropin deficiency became clear when LH plasma values were correlated to the presence of Ad spermatogonia. While both HIR and IIR groups showed normal basal LH levels the LIR group showed LH levels in the hypogonadotropic range. This relative LH deficiency observed in most patients indicates that hypogonadotropic hypogonadism is the main etiologic factor for cryptorchidism. (5).

In summary, concerning the role of elevated temperature and/or hypoxic shock it can only be speculated that pathological changes observed in the low infertility risk group may stem in part from stress caused by elevated temperature. Even if some form of temperature stress would occur, it likely does not impair spermatogonial stem cell development.

## Mini puberty is heat shock resistant

Orchidopexy at the age of about six months has been recommended, based on the assumption that early relocation of the testis into the scrotal environment at the lower temperature will enable germ cells to develop normally and thereby lower the risk of oligospermia and cancer [12]. However, a randomized study investigating the efficacy of GnRHa treatment in restoring defective mini-puberty led to a different conclusion with respect to the effect of elevated temperature on prepubertal testis development [13]. This study showed that gonocytes differentiate into Ad spermatogonia despite cryptorchid position during the treatment with GnRHa [13]. Importantly, a long-term follow-up study showed that infertility in cryptorchidism is prevented by GnRHa treatment, since sperm count was normal in the majority of the treated unilaterally cryptorchid males, while the patients treated with orchidopexy alone became infertile [14]. Furthermore, GnRHa treatment while testes were in the undescended position confirmed that the differentiation into Ad spermatogonia is testosterone- and LH-dependent but temperature-independent [15].

## Heat shock response gene expression is unaltered in cryptorchid testis

Thermosensitive genes are essential for organism to survive exposure to stress [16, 17]. The human heat shock family genes are *HSF1*, *HSF2*, *HSF4*, *HSF5*, *HSFY2*, and *HSFX2* [16, 17]. *HSF1*, *HSFX2*, and *HSF4* expression is identical in testis with or without Ad spermatogonia and does not respond to curative GnRHa treatment (Table 1). Cryptorchid boys whose testes lack Ad spermatogonia belong to the high infertility risk (HIR) group, while patients whose testes contain Ad spermatogonia fall into the low infertility risk (LIR) category. (Table 1) *HSF2* expression is lower in HIR patients who display defective mini-puberty and remains low after six months of GnRHa treatment (Table 1). *HSF5* and *HSFY2* respond positively to GnRHa treatment. *HSF5* is expressed exclusively in the testes and has a critical role in spermatogenesis [18]. Interestingly, the high infertility risk group expresses *HSF5* at a lower level before the treatment (Table 1). Thus, *HSF5* may be involved in the regulation of Ad spermatogonia differentiation.

**Table 1:** RNA sequencing and GeneChip (Affymetrix) data analysis of heat shock factor, heat shock protein and endoplasmic reticulum stress genes. FC; the log-fold changes, FDR; false discovery rate, RPKM; median expression values in reads per kilobase per million for thermosensitive genes before and after GnRHa treatment

Gene	HIR/LIR (RPKM)	log2FC / FDR	before/after GnRHa treatment (RPKM)	log2FC / FDR	Affymetrix probeset identifier (highest variance probe set per gene)	Descended / undescended (HIR + LIR, median log2 signal)	log2FC / FDR
***Heat-shock protein genes***							
***HSP90AA1***	71.2/102.3	−0.53/0.01	75.6/78.1	−0.63/0.01	211968_s_at	10.11/10.26	n.s.
***HSPA1A (HSP70)***	6.58/8.8	−0.40/0.01	7.63/8.66	n.s.	202581_at	8.0/7.4	n.s.
***HSPA2***	1.69/2.49	n.s.	2.6/2.25	−0.61/0.02	211538_s_at	5.33/5.43	n.s.
***HSPA4***	39.6/49.3	−0.28/0.03	44.5/34.5	−0.90/0.0005	208814_at	6.78/6.49	n.s.
***HSPA4L***	20.1/18.0	n.s.	18.15/13.82	−0.90/0.01	205543_at	6.55/7.14	−0.6/0.049
***DNAJB1 (HSP40)***	28.0/34.3	−1.99/0.0006	29.8/27.9	−0.69/0.003	200664_s_at	8.0/8.47	n.s.
***DNAJB2 (HSP40)***	10.9/11.4	n.s.	11.93/11.90	−0.51/0.02	202500_at	6.48/6.83	n.s.
***Endoplasmic reticulum stress genes***							
***ERN1***	1.41/3.15	−0.54/0.02	1.72/3.23	n.s.	207061_at	4.35/4.27	n.s.
***TXNRD1***	17.4/19.1	n.s.	15.3/13.5	−0.79/0.0001	201266_at	8.91/9.03	n.s.
***PRKAR2B***	83/80	n.s.	76.05/53.0	−0.97/0.0006	203680_at	10.9/11.05	n.s.
***CASP3***	8.94/9.23	n.s.	8.53/7.7	−0.72/0.002	202763_at	6.71/6.91	n.s.
***EIF2AK3***	7.38/7.0	n.s.	6.84/6.55	−0.56/0.01	218696_at	7.37/7.64	n.s.
***ATF1***	13.1/12.2	n.s.	12.3/8.3	−0.91/0.0003	1565269_s_at	6.33/6.73	n.s.
***ATF6***	12.49/12.44	n.s.	12.76/10.66	−0.76/0.002	217550_at	7.25/7.4	n.s.
***SKIV2L2***	23.4/27.3	n.s.	23.71/18.3	−0.87/0.0005	1562142_at	3.28/2.95	n.s.
***Heat-shock factor genes***							
***HSF1***	13/14	n.s.	14.5/20.9	n.s.	213756_s_at	4.04/4.26	n.s.
***HSF2***	16.6/24	−0.52/0.0002	16.6/16	−0.82/0.0009	211220_s_at	5.48/5.35	n.s.
***HSF4***	0.81/0.99	n.s.	0.88/1.34	n.s.	210977_s_at	6.01/6.06	n.s.
***HSF5***	0.37/1.0	−1.42/0.0005	0.49/1.43	0.69/0.04	230718_at	3.86/3.46	n.s.
***HSFY2***	0.22/0.22	n.s.	0.17/0.83	1.77/0.0003	224007_at	3.06/3.14	n.s.
***HSFX2***	1.80/1.97	n.s.	2.1/2.91	n.s.	220314_at	5.41/5.24	n.s.

The dramatic upregulation of the heat shock proteins is a key part of the heat shock response and is induced primarily by heat shock factor. Importantly, six out of seven HSP genes analyzed showed no increase in their expression, while *HSPA4L* exhibited weakly significant diminished expression in the descended gonad (Table 1).

Endoplasmic reticulum stress and germ cell apoptosis involve signaling pathways that include eight genes (Table 1) [17]. Their expression increases in response to heat stress [17]. In prepubertal cryptorchid testes, despite elevated temperature, the expression of these genes does not increase (Table 1).

In addition, analysis of our previously published GeneChip expression data [19] including the descended testis as a negative control not exposed to high temperature, showed no differences in the expression of thermosensitive genes, between undescended (HIR and LIR) and descended testes (Table 1). This lack of HSP induction is consistent with the finding that the exposure of undescended testes to abnormally high temperature during prepubertal development does not affect transition into Ad spermatogonia differentiation. Taken together, the expression data for heat-inducible genes argue against a major effect on the temperature stress response in prepubertal cryptorchid testes.

## Conclusion

Current results presented in this focused opinion article are inconsistent with elevated temperature as the major cause of defective transformation of gonocytes into Ad spermatogonia. At the molecular level, there is increasing evidence that argues against heat shock being predominantly responsible for the observed pathological testicular changes. Thus, impaired transformation of gonocytes and spermatogonia into Ad spermatogonia is, in the cases of cryptorchidism as defined in this article, most likely not the result of temperature stress but rather of a severe hormonal imbalance. Bona fide cryptorchidism should therefore typically be considered a serious andrological problem that in most cases cannot be successfully treated by early orchidopexy alone.

## Data Availability

Not applicable.

## Abbreviations

Ad: A dark spermatogonia
ATF1: Activating Transcription Factor 1
ATF6: Activating Transcription Factor 6
CASP3: Caspase 3
DNAJB1 (HSP40): DnaJ Heat Shock Protein Family (Hsp40) Member B1
DNAJB2 (HSP40): DnaJ Heat Shock Protein Family (Hsp40) Member B2
EIF2AK3: Eukaryotic Translation Initiation Factor 2 Alpha Kinase 3
ERN1: Endoplasmic Reticulum to Nucleus Signaling 1
GnRHa: Gonadotropin releasing hormone agonist
HSF1: Heat Shock Transcription Factor 1
HSF2: Heat Shock Transcription Factor 2
HSF4: Heat Shock Transcription Factor 4
HSF5: Heat Shock Transcription Factor 5
HSFX2: Heat Shock Transcription Factor X-Linked 2
HSFY2: Heat Shock Transcription Factor Y-Linked 2
HSP90AA1: Heat Shock Protein 90 Alpha Family Class A Member 1
HSPA1A (HSP70): Heat Shock Protein Family A (Hsp70) Member 1A
HSPA2: Heat Shock Protein Family A (Hsp70) Member 2
HSPA4: Heat Shock Protein Family A (Hsp70) Member 4
HSPA4L: Heat Shock Protein Family A (Hsp70) Member 4-like
HIR: High infertility risk group
LH: Luteinizing hormone
LIR: Low infertility risk group
SRD5A: Steroid 5 Alpha-Reductase **2**
